# T-Cell Composition of the Lymph Node Is Associated with the Risk for Early Rejection after Renal Transplantation

**DOI:** 10.3389/fimmu.2017.01416

**Published:** 2017-10-27

**Authors:** Burç Dedeoglu, Nicolle H. R. Litjens, Annelies E. de Weerd, Frank JMF. Dor, Mariska Klepper, Derek Reijerkerk, Carla C. Baan, Michiel G. H. Betjes

**Affiliations:** ^1^Department of Internal Medicine, Section Nephrology and Transplantation, Erasmus University Medical Center, Rotterdam, Netherlands; ^2^Department of Surgery, Erasmus University Medical Center, Rotterdam, Netherlands

**Keywords:** lymph nodes, T cells, kidney transplantation, rejection, T-cell composition

## Abstract

**Background:**

The T-cell composition within the lymph node (LN) of end-stage renal disease (ESRD) patients differs from the composition within the circulation. Activation of the alloreactive T-cell response within secondary lymphoid organs is important after organ transplantation. However, to date no data are present on LN T-cell subsets and the risk for acute rejection after kidney transplantation.

**Methods:**

T cells from LNs of ESRD patients were analyzed for frequency of recent thymic emigrants, relative telomere length, expression of differentiation markers, and were related to the development of early acute rejection (EAR), occurring within 3 months after renal transplantation (RT). Furthermore, the alloreactive potential of mononuclear cells isolated from the LN and peripheral blood of 10 patients was analyzed. Measures of alloreactive potential included proliferation, cytokine production, frequencies of interferon-gamma-producing cells, and the presence of cytotoxic molecules.

**Results:**

Patients with EAR were younger (*p* = 0.019), cytomegalovirus-seropositive (*p* = 0.037) and usually received dialysis prior to RT (*p* = 0.030). Next to this, patients with EAR showed a lower CD4:CD8 ratio (*p* = 0.027) within the LN. T cells from the LN were similar with regard to alloreactive capacity compared with those within the circulation. Univariate regression analysis showed that the CD4:CD8 ratio (OR: 0.67, *p* = 0.039), patient age (OR: 0.93, *p* = 0.024), and preemptive RT (OR: 0.11, *p* = 0.046) were associated with EAR. After a multivariate analysis, only the CD4:CD8 ratio (OR: 0.58, *p* = 0.019) and preemptive RT (OR:0.05, *p* = 0.012) were associated with EAR.

**Conclusion:**

A lower CD4:CD8 ratio in the LN is associated with a higher risk for the development of rejection within 3 months after RT.

## Introduction

The composition and function of the T-cell compartment in patients prior to renal transplantation (RT) may affect the risk for subsequent acute rejection and infection after transplantation (1–7). For instance, aging-related expansion of highly differentiated T cells within peripheral blood (PB), such as CD8^+^ effector memory CD45RA^+^ (EMRA) T cells or CD4^+^CD28^null^ T cells, is associated with a lower risk for early acute rejection (EAR) after RT (1, 2).

However, T cells within PB are only one of the cell populations that play a role in allograft rejection. Another compartment which may have a significant role in the development of acute rejection is the lymph nodes (LNs). Early after organ transplantation, the direct pathway of alloantigen recognition plays the most important part in the initiation of the alloreactive response (8). One of the key events for this response is migration of donor-derived dendritic cells to LNs of the host, which is followed by activation of alloreactive T cells (8, 9). Next to this, a semi-direct pathway is also being described (8, 10), which involves transfer of intact MHC:peptide complexes from donor antigens presenting cells (APCs) to host APCs, and simultaneous presentation of the processed antigens to CD4^+^ T cells in an indirect manner and the intact complexes to CD8^+^ T cells in a direct manner (10, 11). These pathways show the importance of the T-cell composition within LNs with regard to development of rejection.

The T-cell composition of LN differs substantially from PB as T-cell subsets may or may not be able to migrate to LNs. We and others have studied the T-cell composition of the LN of end-stage renal disease (ESRD) patients and found that highly differentiated T cells were virtually absent from LNs, while they were present within PB (12, 13). Whether the LN T-cell composition and alloreactivity are associated with development of acute rejection after RT has not been studied thus far.

The aim of this study was therefore to investigate whether phenotypical T-cell characteristics within LNs showed an association with development of rejection within 3 months after RT. Furthermore, we have also investigated the alloreactive potential within LN and PB, to assess whether the differences in T-cell composition contributed to this alloreactive potential. The alloreactive potential was assessed by performing mixed lymphocyte reactions (MLRs) and analyzing proliferation, cytokine production, frequencies of interferon-gamma (IFN-γ)-producing cells and presence of cytotoxic molecules within supernatants.

## Materials and Methods

### Study Population

Patients prior to RT in the period from August 2015 to March 2016 were suitable for participation. Each patient gave written informed consent to take part in this study. The study was approved by the Medical Ethical Committee of the Erasmus MC (MEC number 2015-301) and was conducted in accordance with the Declaration of Helsinki and the Declaration of Istanbul.

Clinical variables were assessed as shown in Table 1, including age of the recipient and donor, gender, CMV-seropositivity, human leukocyte antigen (HLA) class I and class II mismatches, current and historical panel reactive antibody (PRA) scores, number of RTs, warm ischemia time, number of living donor transplants, cause of chronic kidney disease, preemptive RT (defined as receiving a kidney before initiation of renal replacement therapy), number of related RT (receiving a kidney from a genetically related donor), and type of rejection. The HLA-typing was assessed according to international standards (American Society for Histocompatibility and Immunogenetics/the European Federation for Immunogenetics) using serologic and DNA-based techniques. The PRAs were determined at the laboratory of the blood bank in Leiden, the Netherlands.

**Table 1:** Patient characteristics.

RT patients (*n* = 38)	No rejection (*n* = 27) (71%)	Rejection (*n* = 11) (29%)	*p*
Age recipient	60 (51–66)	47 (37–58)	**0.019**
Age donor	51 (42–61)	63 (50–69)	0.129
Male gender recipient	17 (63%)	7 (64%)	>0.999
CMV-seropositivity recipient	17 (63%)	11 (100%)	**0.037**
**CMV-serostatus donor/recipient**			
−/−	6 (29%)	0 (0%)	0.154
−/+	7 (26%)	7 (64%)	0.061
+/−	4 (15%)	0 (0%)	0.303
+/+	10 (37%)	4 (36%)	>0.999
Mismatch HLA class I	3 (2–4)	3 (2–4)	0.727
Mismatch HLA class II	1 (1–2)	1 (1–2)	0.824
Mismatch HLA class I and II	4 (4–5)	5 (3–5)	0.949
PRA current (%)	0 (0–4)	0 (0–33)	0.446
PRA historic (%)	4 (0–29)	6 (0–73)	0.727
Number of RT	1 (1–1)	1 (1–1)	>0.999
Warm ischemia time (min)	17 (13–22)	23 (20–27)	**0.045**
Living donor transplant	22 (81%)	8 (73%)	0.667
**Cause of CKD**			
Nephrosclerosis/atherosclerosis/hypertension	6 (22%)	5 (45%)	0.238
Primary glomerulopathies	3 (11%)	2 (13%)	0.615
Diabetes	7 (26%)	0 (0%)	0.084
Polycystic kidney disease	6 (22%)	2 (18%)	>0.999
Other	2 (7%)	1 (9%)	>0.999
Unknown	3 (11%)	1 (9%)	>0.999
Pre-emptive RT	13 (48%)	1 (9%)	**0.030**
Genetically related RT	1 (4%)	1 (9%)	0.501
**Acute rejection type**			
Cellular rejection		7 (64%)	
Antibody-mediated rejection		4 (36%)	

*Data are presented as medians (interquartile range) or as numbers (percentages)*.

*CKD, chronic kidney disease; CMV, cytomegalovirus; HLA, human leukocyte antigen; PRA, panel reactive antibody; RT, renal transplantation*.

Early acute rejection was defined as development of biopsy-proven acute allograft rejection according to the Banff criteria (14, 15) within 3 months after RT.

### PBMC and LNMC Isolation

Peripheral blood mononuclear cells (PBMCs) and lymph node mononuclear cells (LNMCs) were isolated as described previously (12). Briefly, PBMCs were isolated from heparinized blood samples by using Ficoll-Paque Plus (GE healthcare, Uppsala, Sweden). Blood was drawn from renal transplant recipients 1 day before RT.

The waste material from renal transplant recipients removed during the implantation of the renal allograft was used to collect iliac LNs. The LNs were cut into small pieces and transferred onto a 70-µm sieve. A cell suspension was obtained after mashing the small pieces through this sieve. The cell suspension was then washed multiple times to eliminate remaining fat tissue. The isolated PBMCs and LNMCs were stored at −150°C with a minimum amount of 10 × 10^6^ cells/vial. Median yield of the LN amounted to 20 × 10^6^ cells.

### T-Cell Differentiation Status

Whole blood and freshly isolated LNMCs were used to assess T-cell differentiation status by flowcytometry. Differentiation status was based upon the study by Sallusto et al. (16), as described in detail previously (17). Briefly, expression of CD45RO and CCR7 was used to determine naïve and memory T-cell populations. Memory T-cell subsets were further defined as central memory (CM) T cells (CD45RO^+^CCR7^+^ T cells), effector memory (EM) T cells (CD45RO^+^CCR7^−^ T cells), and EMRA T cells (CD45RA^+^CCR7^−^ EM T cells) (16). In addition, CD28 expression was analyzed as this is lost upon T-cell differentiation (18–21). Cells were stained as described previously (2), and were measured on the FACSCanto II (BD) and analyzed with the fluorescence-activated cell sorting (FACS) Diva software version 6.1.2 (BD).

To dissect T cells into early- and late-differentiated cells, expression of CD28, CD27, CD57, and PD-1 was also analyzed. Naïve and CM T cells express CD28 and CD27. EM and EMRA T cells progressively lose expression of these molecules [CD4^+^ T cells first lose CD27 and then CD28, while the opposite is true for CD8^+^ T cells (22–24)] and express CD57 and PD-1 (25–28). Thus, early-differentiated T cells were defined as CD28^+^CD27^+^, CD28^+^PD-1^−^, and CD28^+^CD57^−^ T cells, while CD28^null^CD27^−^, CD28^null^PD-1^+^, and CD28^null^CD57^+^ T cells were defined as late-differentiated T cells. This measurement was performed on the Navios flow cytometer (Beckman Colter) and collected data were evaluated with the Kaluza software version 1.3 (Beckman Colter). T cells were analyzed with DuraClone IM T-cell subsets tubes (Beckman Colter) as described previously (12).

### Relative Telomere Length (RTL)

Relative telomere length of CD4^+^ and CD8^+^ T cells was determined by using flow fluorescent *in situ* hybridization on thawed PBMCs and LNMCs, as described in detail previously (17).

### Assessment of RTEs Using CD31 and TREC Content

Recent thymic emigrants (RTEs) were defined as naïve T cells expressing CD31 and were assessed by flow cytometry, as described previously (29). T-cell receptor excision circle (TREC) content was determined using 1 × 10^6^ snap-frozen PBMCs and LNMCs. DNA was isolated from these snap-frozen samples and TREC content was detected using quantitative polymerase chain reaction as described previously (30). The TREC content is depicted as 1/ΔCT.

### Allogeneic Stimulation

Peripheral blood mononuclear cells and lymph node mononuclear cells from renal transplant recipients (responders) were thawed and rested overnight. Then PBMCs and LNMCs were labeled with carboxyfluorescein succinimidyl ester (CFSE) (Molecular Probes^®^, Leiden, the Netherlands) according to manufacturer’s instructions and stimulated in triplicate at 5 × 10^4^/well with irradiated PBMCs (40 Gy) of their corresponding donor, at a 1:1 ratio for 6 days. As a negative control, responders were stimulated with their own irradiated PBMCs or LNMCs (autologous stimulation). Responder cells were stimulated with phytohemagglutinin (PHA) 5 µg/ml to examine their maximum proliferative potential. On day 6, wells of the same condition were pooled and supernatant stored at −80°C. Proliferation was analyzed by measuring CFSE dilution and determining the frequency of CFSE^−^ cells. For this purpose, cells were stained using the following antibodies: AmCyan-labeled anti-CD3 (BD), pacific blue (PacB)-labeled anti-CD4 (BD), APC-Cy7-labeled anti-CD8 (BD), phycoerythrin (PE)-Cy7-labeled anti-CCR7 (BD Pharmigen), APC-labeled anti-CD45RO (BD), and PE-labeled anti-CD28 (BD). A dump-channel was applied to exclude unwanted cells from the analysis, by co-staining cells for the live-dead marker 7-AAD, peridin chlorophyll protein (PerCP)-labeled anti-CD19 (BD), PerCP-Cy5.5-labeled anti-CD56 (Biolegend), and PerCP-labeled anti-CD14 (BD) (Figure S1 in Supplementary Material). Samples were measured on the FACSCanto II (BD) and analyzed using FACS Diva software version 6.1.2 (BD).

### Analysis of Cytokine and Granzyme B Production

Concentrations of IFN-γ, tumor necrosis factor-alpha (TNF-α), and granzyme B were determined from collected supernatants. These supernatants were analyzed with the human cytometric bead array (CBA) flex set (BD) according to manufacturer’s instructions. Briefly, a standard curve for each analyte using a four-parameter logistic regression analysis was created. This curve was based upon standards with fixed concentrations of each analyte and their corresponding median fluorescence intensities (MFIs). Then, MFIs of the various analytes within the samples were converted to concentrations (pg/mL). Samples were measured on the FACS Canto II (BD) and concentrations were determined with GraphPad Prism 5 (CA, USA).

### IFN-γ ELISPOT Assay

Frequencies of IFN-γ-producing cells (spots/100,000 cells) following autologous, allogeneic, or PHA stimulation were measured with an Enzyme-Linked ImmunoSpot (ELISPOT) assay (U-CyTech, Utrecht, The Netherlands). During the day 1, the ELISPOT plate was coated with the antibody and incubated overnight. The same day cells were thawed and rested overnight. The following day, the assay plate was blocked using a blocking buffer and incubated for 1 h at 37°C. After the plate was washed with phosphate-buffered saline (PBS), cells were pipetted into wells and stimulated in triplicate, as described earlier, for 1 day. Thereafter, plates were washed first with PBS and then with PBS-Tween. Spots were made visible according to manufacturer’s instructions. Spots were analyzed using the ELISpot reader (Bioreader^®^-600V, BIO-SYS GmbH, Karben, Germany).

### Statistical Analysis

Variables are presented as medians with interquartile ranges. Differences between paired samples (PB and LN from the same patient) were analyzed using the Wilcoxon signed-rank test. Differences between continuous variables from two independent groups were assessed with the Mann–Whitney *U* test. Differences between categorical variables were analyzed either with the Pearson’s chi-squared test or with the Fisher’s exact test depending on the expected values in any of the cells of a contingency table. Associations between rejection and the assessed parameters were analyzed using a binary logistic regression analysis. The significance level (*p*-value) was two-tailed and a value of α = 0.05 was used. Statistical analyses were performed using SPSS^®^ version 21.0 for Windows^®^ (SPSS Inc., IL, USA) and GraphPad Prism 5 (CA, USA). Figures were created with GraphPad Prism 5 (CA, USA).

## Results

### Patient Characteristics

Patient characteristics (*n* = 38) are shown in Table 1. Median patient age was 58 years and median donor age was 55 years. Most of the patients underwent an RT for the first time (82%), while 18% underwent a transplantation for the second time. Most common cause of ESRD was nephrosclerosis/atherosclerosis/hypertension (29%) followed by polycystic kidney disease (21%), together accounting for half of the cases. Overall, 24 (63%) patients received dialysis treatment prior to RT. Eleven of the 38 patients (29%) developed an EAR. Majority of the rejections were classified as cellular rejection (64%) and the remaining as antibody-mediated rejection. Median patient age was significantly younger within the rejectors compared with the non-rejectors (47 vs. 60, *p* < 0.019). All patients who developed an EAR were CMV-seropositive, while 63% of the non-rejectors was CMV-seropositive (*p* = 0.037). Median warm ischemia time was longer for the rejectors compared with the non-rejectors (23 min vs. 17 min, *p* = 0.045). Majority of the rejectors had dialysis prior to RT (91%), while 52% of the non-rejectors received this therapy (*p* = 0.030). Immunological risk factors like PRA score, HLA mismatches, or unrelated donor transplantation were not different between the two groups.

### T-Cell Composition of the LN Prior to RT Showing an Association with EAR

**Figure 1:**
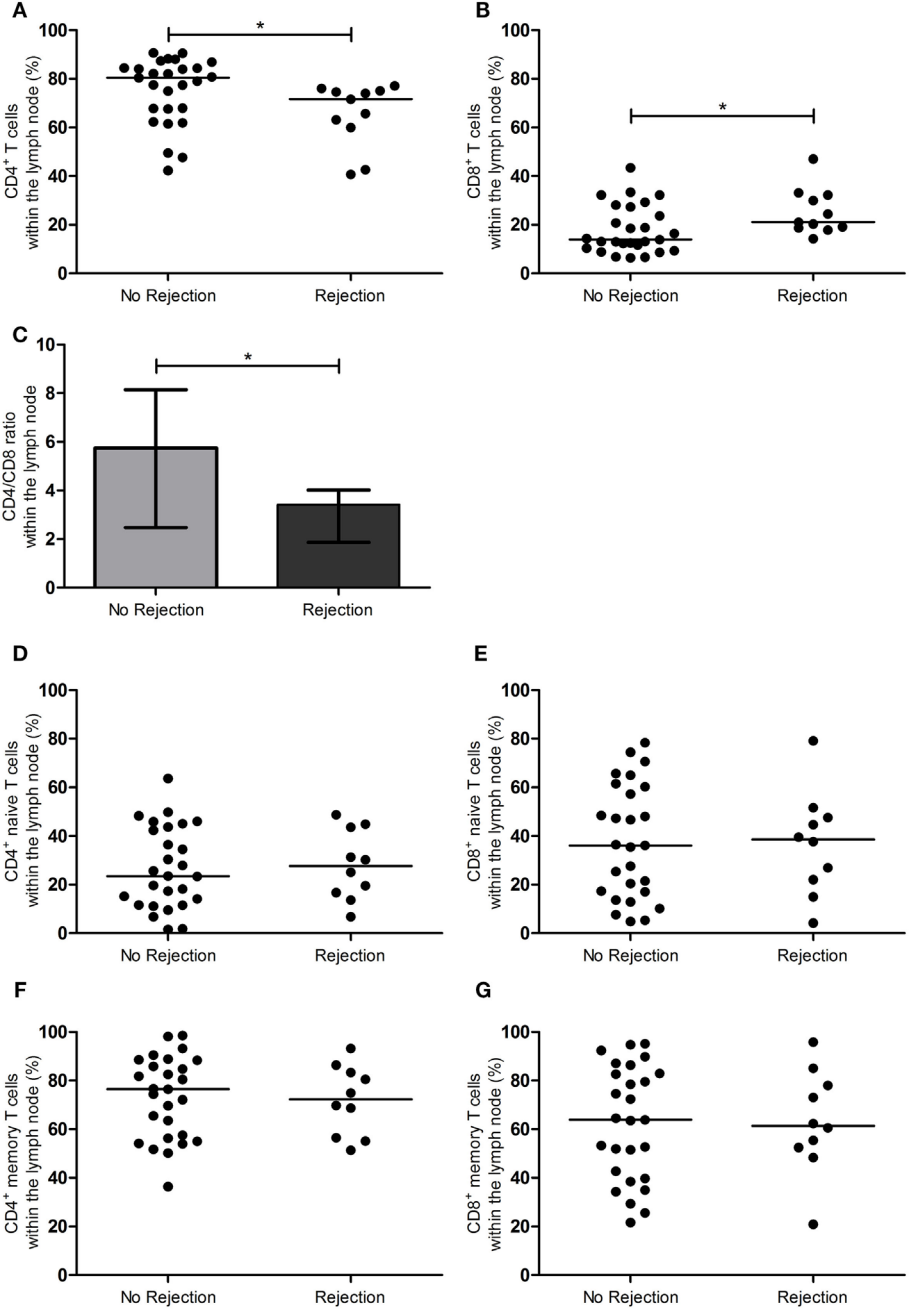
CD4^+^ and CD8^+^ T-cell subsets in the lymph node between rejection and no rejection groups. **(A)** Frequencies of CD4^+^ T cells and **(B)** CD8^+^ T cells, **(C)** CD4:CD8 ratio, **(D)** frequencies of CD4^+^ naïve T cells, **(E)** CD8^+^ naïve T cells, **(F)** CD4^+^ memory T cells, and **(G)** CD8^+^ memory T cells within the lymph nodes are shown between the no rejection and rejection groups. Data are presented with individual percentages and medians or bars with interquartile range and medians. Significant differences were calculated and shown (*p < 0.05, **p ≤ 0.01, ***p ≤ 0.001).

**Figure 2:**
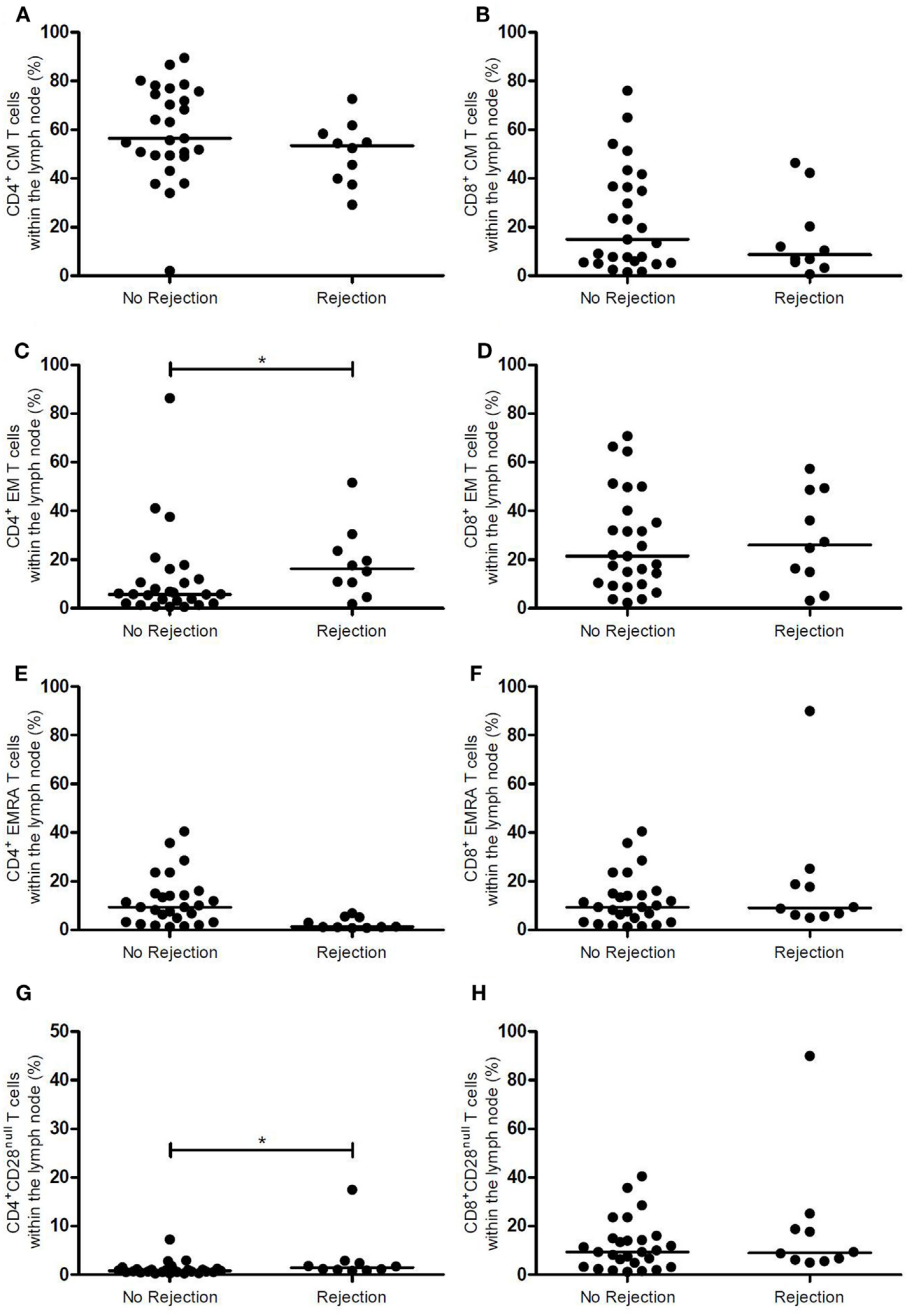
Memory CD4^+^ and CD8^+^ T-cell subsets and CD28 expression in the lymph node between rejection and no rejection groups. **(A)** Frequencies of CD4^+^ CM T cells, **(B)** CD8^+^ CM T cells, **(C)** CD4^+^ EM T cells, **(D)** CD8^+^ EM T cells, **(E)** CD4^+^ EMRA T cells, **(F)** CD8^+^ EMRA T cells, **(G)** CD4^+^CD28^null^ T cells, and **(H)** CD8^+^CD28^null^ T cells within the lymph nodes are shown between the no rejection and rejection groups. Data are presented with individual percentages and medians. Significant differences were calculated and shown (*p < 0.05, **p ≤ 0.01, ***p ≤ 0.001). CM, central memory; EM, effector memory; EMRA, effector memory CD45RA^+^.

Figures 1 and 2 show the T-cell differentiation status of CD4^+^ and CD8^+^ T cells within the LNs of the rejectors and non-rejectors. Median frequency of CD4^+^ T cells prior to RT is significantly lower (*p* = 0.014) and median frequency of CD8^+^ T cells is significantly higher (*p* = 0.019) within the rejectors (Figures 1A,B). This led to a significantly lower CD4:CD8 ratio in LNs of patients with EAR (3.4 vs. 5.7, *p* = 0.027) (Figure 1C). Furthermore, median frequencies of CD4^+^ EM T cells and CD4^+^CD28^null^ T cells were significantly higher within the rejectors (*p* = 0.044 and *p* = 0.008, respectively) (Figures 2C,G). But overall, median percentage of CD4^+^CD28^null^ T cells remained low in both groups (0.8 vs. 1.5%). No other significant differences were observed for the other CD4^+^ or CD8^+^ T-cell subsets.

Assessment of T-cell differentiation status can be further refined by differential expression levels of CD27, CD57, and PD-1 in combination with CD28. However, this approach did not show any differences between the two groups (Table 2).

**Table 2:** CD4^+^ and CD8^+^ T-cell differentiation status in lymph node with regard to the development of rejection.

	*n*	No rejection	*n*	Rejection	*p*
**CD4^+^**					
CD4^+^CD28^+^CD27^+^ (%)	25	93.8 (91.6–96.6)	8	93.8 (81.1–94.7)	0.578
CD4^+^CD28^null^CD27^−^ (%)	25	0.3 (0.1–0.6)	8	0.2 (0.1–0.8)	0.951
CD4^+^CD28^+^PD-1^−^ (%)	25	73.5 (64.0–82.0)	8	76.3 (72.0–80.4)	0.665
CD4^+^CD28^null^PD-1^+^ (%)	25	0.4 (0.2–0.9)	8	0.5 (0.3–1.2)	0.578
CD4^+^CD28^+^CD57^−^ (%)	25	97.6 (95.3–98.6)	8	97.0 (96.5–97.7)	0.272
CD4^+^CD28^null^CD57^+^ (%)	25	0.1 (0.0–0.2)	8	0.1 (0.0–0.2)	0.885
**CD8^+^**					
CD8^+^CD28^+^CD27^+^ (%)	25	89.6 (84.1–93.3)	8	90.4 (78.8–92.2)	0.789
CD8^+^CD28^null^CD27^−^ (%)	25	1.9 (1.0–2.6)	8	1.7 (1.0–6.5)	0.789
CD8^+^CD28^+^PD-1^−^ (%)	25	62.2 (49.5–75.1)	8	66.9 (45.8–75.1)	0.853
CD8^+^CD28^null^PD-1^+^ (%)	25	2.4 (1.6–5.2)	8	2.6 (1.6–4.8)	0.951
CD8^+^CD28^+^CD57^−^ (%)	25	87.4 (80.2–91.0)	8	86.3 (75.5–88.8)	0.374
CD8^+^CD28^null^CD57^+^ (%)	25	1.5 (1.0–2.3)	8	1.5 (0.9–4.3)	0.757

*Data are presented as medians (interquartile range)*.

In summary, a lower CD4:CD8 ratio was associated with rejection.

### RTL, RTEs, and TREC Content in the LN Not Associated with Rejection

**Figure 3:**
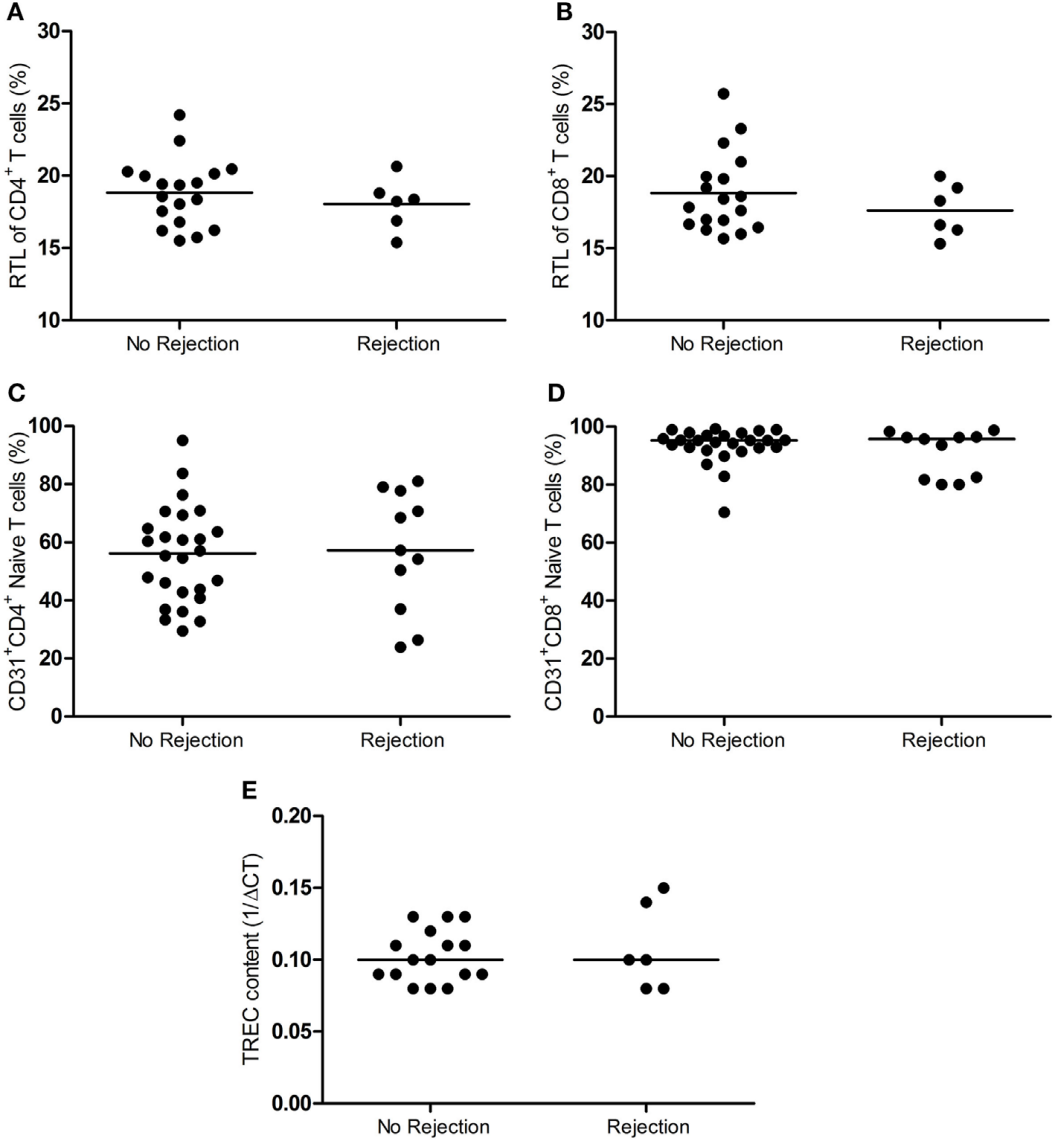
RTL, RTE frequency and TREC content in the lymph node between rejection and no rejection groups. The RTL of **(A)** CD4^+^ T cells and **(B)** CD8^+^ (T cells) within the lymph nodes between the no rejection and rejection groups are shown. Frequencies of **(C)** CD4^+^ and **(D)** CD8^+^ RTEs (CD31^+^ naïve T cells) within the lymph nodes between the no rejection and rejection group are shown. **(E)** The TREC content (1/ΔCT) within the lymph nodes between the no rejection and rejection group is shown. Data are shown as individual points and medians. Significant differences were calculated and shown (*p < 0.05, **p ≤ 0.01, ***p ≤ 0.001). RTEs, recent thymic emigrants; RTL, relative telomere length; TREC, T-cell receptor excision circle.

The telomere length is indicative of T-cell replicative history, while CD31 expression on naïve T cells and TREC content are a measure of thymic output of naïve T cells. These parameters are strongly associated with the age and therefore a measure of the biological age of the T-cell system (31–36). Figure 3 demonstrates the RTL of T cells, RTE frequency, and the TREC content in LNs between the rejectors and non-rejectors. RTL of CD4^+^ and CD8^+^ T cells was similar between the two groups (Figures 3A,B). Also, no differences were observed for CD4^+^ and CD8^+^ RTEs between rejection and no rejection (Figures 3C,D). Finally, the TREC content was also not associated with development of rejection (Figure 3E).

### A Lower CD4:CD8 Ratio within the LN and Dialysis Prior Transplantation Associated with Rejection

Table 3 shows a univariate regression analysis of the significant clinical and immunological parameters with development of EAR. This univariate analysis showed that a higher CD4:CD8 ratio was associated with a lower risk for EAR (OR = 0.67, *p* = 0.039). Next to this, an older patient age and preemptive RT were also associated with a lower risk for rejection (OR = 0.93, *p* = 0.024; OR = 0.11, *p* = 0.046, respectively). Then these parameters were put into a multivariate regression analysis for a first model (Table 4). This showed that age was not associated with development of EAR. On the other hand, a higher CD4:CD8 ratio showed a tendency for a lower risk for rejection (OR = 0.63, *p* = 0.053), while preemptive RT still showed a significant association with development of EAR (OR = 0.06, *p* = 0.023). Taking the parameter “age” out of the equation showed a second model with significant associations (Table 4). A higher CD4:CD8 ratio and preemptive RT were both associated with a lower risk for EAR (OR = 0.58, *p* = 0.019; OR = 0.05, *p* = 0.012, respectively).

**Table 3:** Univariate regression analysis between rejection and immunological and clinical parameters.

	OR	95% CI	*p*
CD4^+^ T cells (%)	0.95	0.90–1.00	0.062
CD4^+^ EM T cells (%)	1.02	0.98–1.06	0.314
CD4^+^CD28^null^ T cells (%)	1.30	0.86–1.95	0.216
CD4:CD8 ratio	0.67	0.45–0.98	**0.039**
CD8^+^ T cells (%)	1.08	1.00–1.16	0.057
Age recipient	0.93	0.88–0.99	**0.024**
CMV-seropositivity recipient	0.00	0.00–N/A	0.999
Warm ischemia time (min)	1.06	0.96–1.16	0.241
Preemptive RT	0.11	0.01–0.96	**0.046**

*CMV, cytomegalovirus; EM, effector memory; RT, renal transplantation; N/A, not available*.

**Table 4:** Multivariate regression analysis between rejection and immunological and clinical parameters.

	OR	95% CI	*p*
**Model 1**			
CD4:CD8 ratio	0.63	0.39–1.01	0.053
Age recipient	0.97	0.90–1.04	0.393
Preemptive RT	0.06	0.01–0.68	**0.023**
**Model 2**			
CD4:CD8 ratio	0.58	0.36–0.92	**0.019**
Preemptive RT	0.05	0.00–0.51	**0.012**

*RT, renal transplantation*.

Thus, a lower CD4:CD8 ratio in the LN and receiving dialysis prior to transplantation increases the risk for the development of rejection after transplantation.

### T Cells from the LN and PB Proliferating Similarly upon Allogeneic Stimulation

**Figure 4:**
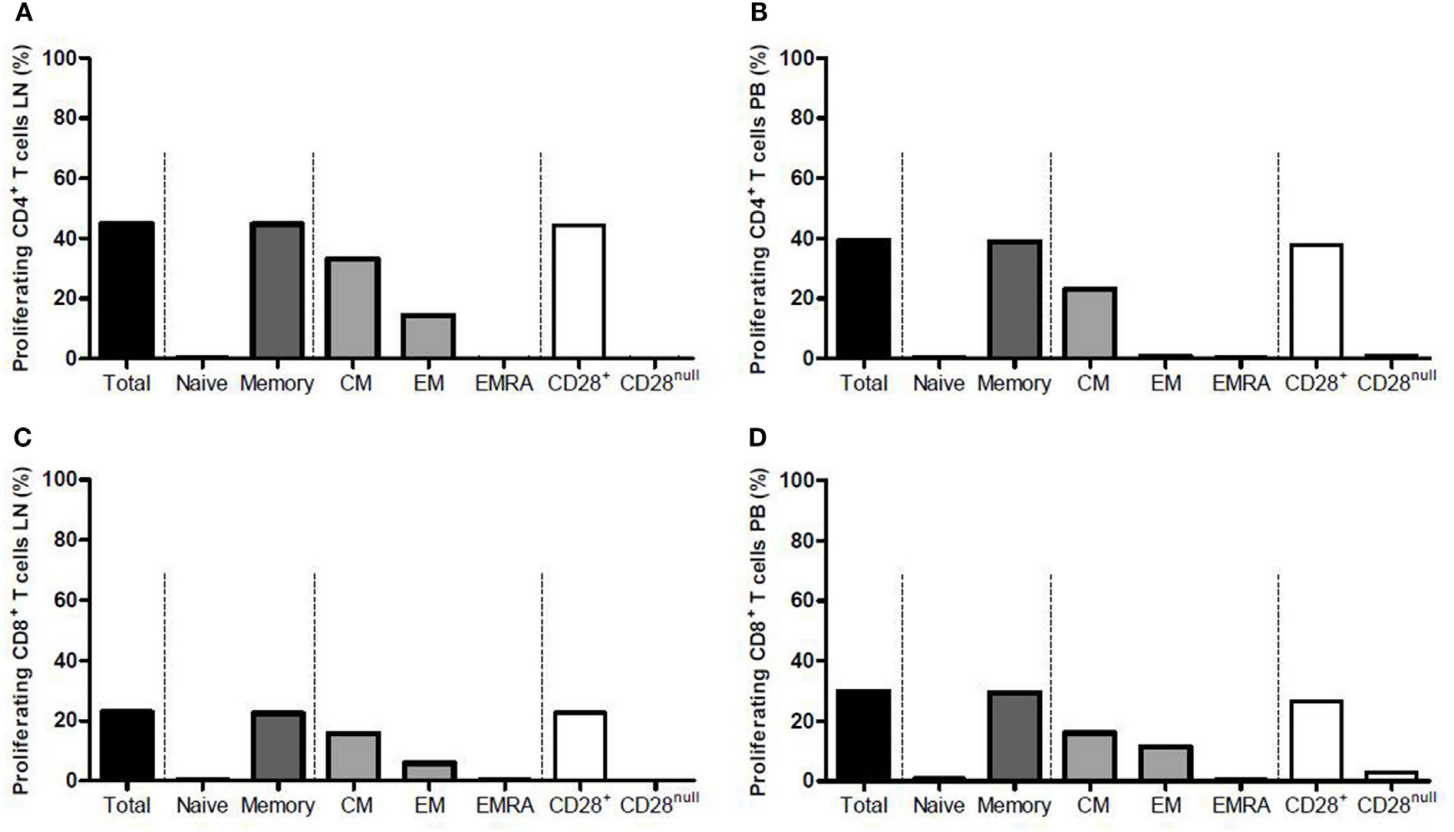
Proliferation of CD4^+^ and CD8^+^ T cells from the lymph node and peripheral blood after allogeneic stimulation. Frequencies of proliferating CD4^+^ T cells within **(A)** the lymph node and **(B)** the peripheral blood are shown after allogeneic stimulation. Frequencies of proliferating CD8^+^ T cells within **(C)** the lymph node and **(D)** the peripheral blood are shown after allogeneic stimulation. Each graph is divided into four parts marked with the dotted lines. From left to right: the total proliferating T-cell population is shown, which then is subdivided into naïve and memory T cells in the second portion, the third portion shows the subdivision of the memory T-cell population into CM, EM, and EMRA T cells, and the fourth portion shows the subdivision of the total T-cell population into CD28^+^ and CD28^null^ T cells. Frequencies are presented with bars and medians. CM, central memory; EM, effector memory; EMRA, effector memory CD45RA^+^.

Median frequency of proliferating CD4^+^ T cells from the LN rose significantly from 2.4 to 45.0% (*p* = 0.002) upon allogeneic stimulation (Figure 4A), and that of CD4^+^ T cells from the PB increased from 2.1 to 39.3% (*p* = 0.004) (Figure 4B). Within the proliferating CD4^+^ T cells, majority of the cells were memory T cells (mainly CM) (Figures 4A,B). The CD28^+^ T-cell fraction was the main proliferating fraction within proliferating CD4^+^ T cells in PB and LN (>99.0% proliferation in both compartments) (Figures 4A,B).

The CD8^+^ T cells showed similar results compared with the CD4^+^ T cells. Median frequency of proliferating CD8^+^ T cells from the LN rose significantly from 1.4 to 22.9% (*p* = 0.004) upon allogeneic stimulation (Figure 4C) and median frequency of CD8^+^ T cells from PB increased from 0.6 to 29.9% (*p* = 0.002) (Figure 4D). CD8^+^ memory T cells comprised the largest fraction within proliferating CD8^+^ T cells in both LN and PB, with CM T cells also being the major contributor within this fraction (Figures 4C,D). The CD28^+^ T-cell fraction was again the main proliferating fraction within proliferating CD8^+^ T cells in LN and PB (Figures 4C,D).

Comparing the frequency of proliferating T cells upon alloreactive stimulation between LN and PB showed no differences except for CD8^+^CD28^null^ T cells within proliferating CD8^+^ T cells. Median frequency of these cells within LN amounted to 0.3%, while this was 2.9% within PB (*p* = 0.037), which is probably due to the higher presence of these cells within PB compared with LN.

Polyclonal stimulation with PHA induced >95% proliferation of CD4^+^ T cells and CD8^+^ T cells from both compartments, although this response was significantly higher in CD4^+^ and CD8^+^ T cells from LN (*p* = 0.037 for both subsets). The functional assays could not be performed in all patients as the yield of cells from the LN was not sufficient enough in some cases. For this reason, the number of patients with EAR included in this part of the study was too low (*n* = 3) for adequate comparison with patients without EAR.

In conclusion, the alloreactive potential is not different between T cells from LN and PB, but T cells from LN show a slightly higher proliferative potential in response to PHA.

### IFN-γ ELISPOT Showing No Differences between LN and PB, but IFN-γ and Granzyme B Production by LNMCs Lower than PBMCs after 6 Days of Allogeneic Stimulation

**Figure 5:**
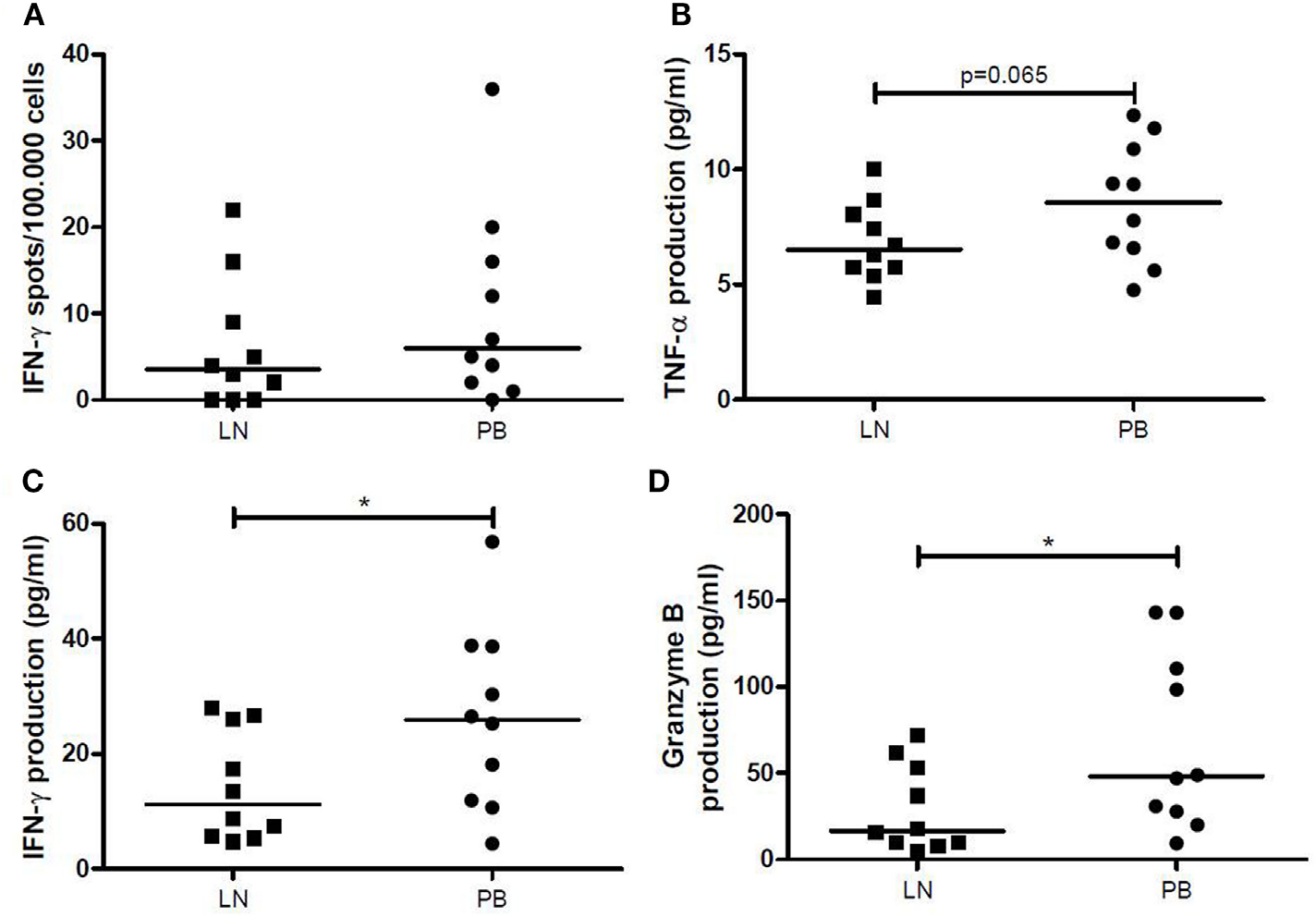
IFN-γ, TNF-α, and granzyme B production after allogeneic stimulation. **(A)** The number of IFN-γ spots per 100,000 cells by LNMCs and PBMCs after allogeneic stimulation for 1 day is shown. The production of **(B)** TNF-α, **(C)** IFN-γ, and **(D)** granzyme B (pg/mL) by LNMCs and PBMCs after allogeneic stimulation for 6 days is shown. Data are shown as individual points and medians. Significant differences were calculated and shown (*p < 0.05, **p ≤ 0.01, ***p ≤ 0.001). IFN-γ, interferon-gamma; LNMCs, lymph node mononuclear cells; PBMCs, peripheral blood mononuclear cells; TNF-α, tumor necrosis factor-alpha.

Frequency of IFN-γ-producing LNMCs increased significantly after allogeneic stimulation (*p* = 0.034), while a similar tendency was seen for PBMCs (*p* = 0.083). However, median amount of spots remained low after allogeneic stimulation (3.5/1 × 10^5^ for the LN and 6/1 × 10^5^ for the PB). Furthermore, there were no significant differences between the two different compartments after allogeneic stimulation (Figure 5A). Stimulation with PHA showed a significantly higher IFN-γ secretion by PBMCs compared with T cells within LNMCs (256/1 × 10^5^ vs. 126/1 × 10^5^, *p* = 0.047).

After allogeneic stimulation of LNMCs and PBMCs for 6 days, concentrations of TNF-α, IFN-γ and granzyme B in the supernatants were significantly increased. LNMCs had a lower production of TNF-α (*p* = 0.065) (Figure 5B), IFN-γ, and granzyme B compared with PBMCs (*p* = 0.020 and *p* = 0.049, respectively) (Figures 5C,D). Interestingly, amount of IFN-γ spots after allogeneic stimulation for 1 day correlated significantly with production of IFN-γ by LNMCs as well as PBMCs after 6 days (ρ = 0.88, *p* = 0.001 and ρ = 0.64, *p* = 0.048, respectively).

Thus, production of TNF-α, IFN-γ, and granzyme B by LNMCs and PBMCs increases after allogeneic stimulation. However, generation of these products is lower by LNMCs than by PBMCs.

## Discussion

This study has investigated the relation between T-cell characteristics of the LN and acute rejection after RT for the first time. Surprisingly, a lower CD4:CD8 ratio in LNs was significantly associated with a higher risk for rejection. Studies have shown that CD8^+^ T cells play an important role in allograft rejection (37, 38). Antigen presenting cells can present the alloantigen to CD4^+^ and CD8^+^ T cells that are present within the LN, which will lead to activation of these cells. Having a lower CD4:CD8 ratio might tip the balance toward a more cytotoxic profile, which could promote allograft rejection.

A study by Ford et al. has shown in a murine skin graft model that a high initial frequency of antigen-specific CD8^+^ T cells resulted in development of CD8^+^ T cells which were able to produce multiple cytokines and were more prone to escape co-stimulation blockade (39). Next to this, a study by Shenoy et al. showed that a lower CD4:CD8 ratio in bronchus-associated lymphoid tissue was associated with a higher frequency of acute rejections within 1 year after lung transplantation (40). Thus, having a high initial frequency of CD8^+^ T cells in LNs could lead to a higher chance for presence of alloreactive CD8^+^ T cells with increasing the risk for rejection.

It is known that alloantigen recognition can occur *via* direct or indirect pathways (8), but next to this a semi-direct pathway is also being described (8, 10). This semi-direct pathway involves transfer of intact MHC:peptide complexes from donor APCs to host APCs and also the processing of antigens by host APCs (10). The host APCs can then simultaneously present the processed antigens to CD4^+^ T cells in an indirect manner, while they present the intact complexes to CD8^+^ T cells in a direct manner (10, 11). At the same time, CD4^+^ T cells can provide help to CD8^+^ T cells to activate them (10). It has been shown that CD4^+^ T cells which are activated by the indirect pathway can successfully enhance direct pathway CD8^+^ T-cell responses (41). As the direct pathway is the most important pathway in the initiation of alloreactive responses (8), a higher frequency of CD8^+^ T cells within LNs prior to transplantation will increase the possibility for allorecognition by the direct and also the semi-direct pathway.

Next to this, we have found that younger patients have a higher risk for EAR. Even though this finding was not significant after a multivariate analysis, it still showed an important difference between the rejectors and non-rejectors after a univariate analysis. This association between a younger age and an increased risk for rejection has also been found by previous studies (42, 43). An aged immune system in the older patients may underlie the reduced risk for rejection. In addition, ESRD patients undergo uremia-associated premature aging of their T-cell system (17, 29, 30), which further impairs the alloreactivity of their immune system. For this reason, we have studied in detail the relation between several immune parameters of T-cell aging and clinical outcome after RT. In particular, the increased presence of highly differentiated CD8^+^ EMRA T cells or CD4^+^CD28^null^ T cells in the circulation was associated with a decreased risk for acute rejection but no other parameters such as telomere length and thymic output of naïve T cells (1, 2). In this study, we did not find an association between all tested immune parameters of T-cell aging and acute rejection. The absence of highly differentiated T cells within the LN is most likely the reason that we also could not relate the presence of these cells to acute rejection (12).

Furthermore, we analyzed differences in IFN-γ production by mononuclear cells isolated from LN and PB using the ELISPOT assay. Previous studies have shown that this assay shows important correlations with regard to rejection and graft function after RT (44–47). However, we saw no differences with regard to IFN-γ-producing cells from both compartments. On the other hand, we saw that production of IFN-γ and granzyme B was higher by PBMCs after 6 days of stimulation. Again, this is probably due to the increased presence of more differentiated cells within PB compared with LN (12). Next to this, proliferation of T cells upon stimulation with alloantigens was also similar between the two compartments. This means that LNMCs have a similar alloreactive potential, with the LNMCs showing a lower production of cytokines and cytotoxic molecules, due to the presence of less differentiated T cells within LNs.

There are some limitations to this study. We could not analyze functional differences between rejectors and non-rejectors. This was due to the lack of material from the rejectors, which made a proper comparison between the two groups difficult. However, we believe that the functional analyses within both compartments also give significant insight into the impact of compositional differences between the LN and PB, which has also not been investigated before. Next to this, we did not analyze frequencies of regulatory T cells (Tregs). This was not done as the current study was designed as a follow-up on our previous work, in which we have analyzed T-cell aging parameters in the PB in relation to acute rejection after kidney transplantation. Furthermore, the usage of FoxP3 expression to identify Tregs can also lead to the identification of activated CD4^+^ T cells (48). For a proper analysis of Tregs, their suppressive properties need to be demonstrated together with the demethylation of the FoxP3 gene which requires more cells than harvested in general from the LNs.

This study is the first which shows an association between the T-cell CD4:CD8 ratio of the LN and development of EAR after RT. Although this finding is of considerable interest, it needs to be validated in a study with a larger cohort. However, the results show that the LN should be considered as a different compartment with its own contribution to the risk for the development of rejection. Although speculative, one of the implications of our findings could be that highly differentiated memory T cells in the PB are relevant for and associated with acute rejection as they can directly enter peripheral tissues such as the renal transplant. In contrast, these cells cannot enter the LN and alloreactivity in this environment is stimulated by donor-derived APC interacting with naïve and CM T cells. The relative contribution of both pathways needs to be further elucidated.

In conclusion, a lower CD4:CD8 ratio within LN is associated with a higher risk for development of rejection within 3 months after RT.

## Ethics Statement

The study was approved by the Medical Ethical Committee of the Erasmus MC (MEC number 2015-301) and was conducted in accordance with the Declaration of Helsinki and the Declaration of Istanbul. Each patient voluntarily signed an informed consent to participate in this work.

## Author Contributions

BD, NL, MK, and DR performed experiments. BD, NL, MK, and DR analyzed the results and made the figures. AW, MB, and NL designed the research. BD, NL, AW, FD, MK, DR, CB, and MB wrote and revised the paper.

## Conflict of Interest Statement

The authors declare that the research was conducted in the absence of any commercial or financial relationships that could be construed as a potential conflict of interest.

## Supplementary Material

Figure S1 Analysis of proliferation after allogeneic stimulation of PBMCs and LNMCs. An example of the gating strategy is shown. The left column represents the analysis of LNMCs, while the right column shows the analysis of PBMCs after allogeneic stimulation. From top to bottom: first, lymphocytes were selected based on the forward and sideward scatter. Then, living CD3^+^ T cells were selected from these lymphocytes. The CD3^+^ T cells were then further divided into CD4^+^ and CD8^+^ T cells. Each population was then further dissected into different subsets using CCR7 and CD45RO. Only CD4^+^ T cells are shown, but the same analysis was applied on CD8^+^ T cells. The bottom graphs show an example of the analysis of proliferation after allogeneic stimulation with CFSE dilution. CFSE, carboxyfluorescein succinimidyl ester; LNMCs, lymph node mononuclear cells; PBMCs, peripheral blood mononuclear cells.
