# Supplementary figures and images for: T-Cell Composition of the Lymph Node Is Associated with the Risk for Early Rejection after Renal Transplantation
Source: Front Immunol. 2017 Oct 27;8:1416. doi: 10.3389/fimmu.2017.01416 (PMC5663687; doi:10.3389/fimmu.2017.01416)

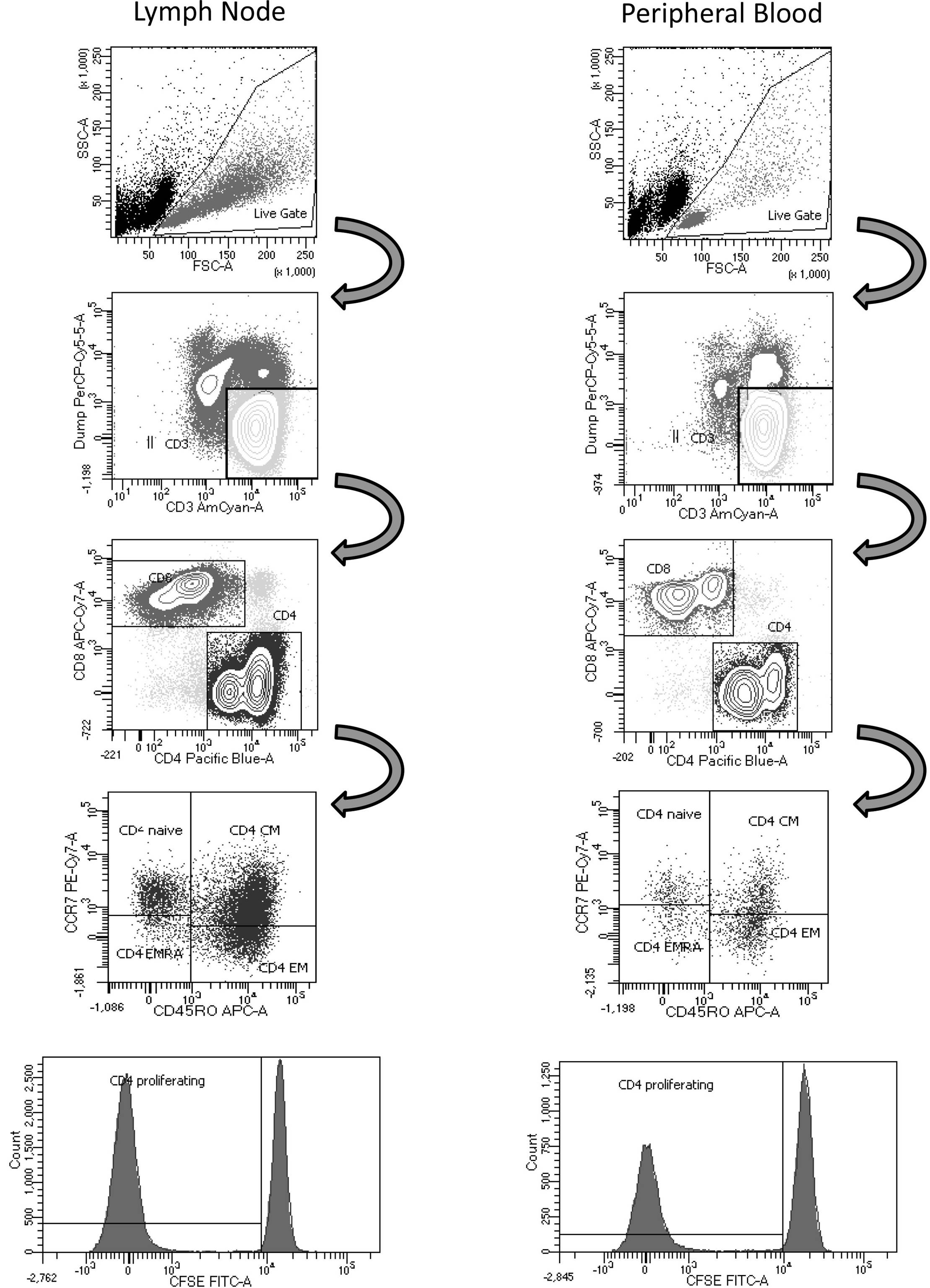

Supplement: Figure S1 — Analysis of proliferation after allogeneic stimulation of PBMCs and LNMCs. An example of the gating strategy is shown. The left column represents the analysis of LNMCs, while the right column shows the analysis of PBMCs after allogeneic stimulation. From top to bottom: first, lymphocytes were selected based on the forward and sideward scatter. Then, living CD3+ T cells were selected from these lymphocytes. The CD3+ T cells were then further divided into CD4+ and CD8+ T cells. Each population was then further dissected into different subsets using CCR7 and CD45RO. Only CD4+ T cells are shown, but the same analysis was applied on CD8+ T cells. The bottom graphs show an example of the analysis of proliferation after allogeneic stimulation with CFSE dilution. CFSE, carboxyfluorescein succinimidyl ester; LNMCs, lymph node mononuclear cells; PBMCs, peripheral blood mononuclear cells. [file image_1.jpeg]
